# Commissioning and robustness evaluation of external laser sensor for respiratory gating in carbon‐ion radiotherapy

**DOI:** 10.1002/acm2.70438

**Published:** 2025-12-29

**Authors:** Tae‐Ho Kim, Soorim Han, Dohyeon Yoo, Sangmin Lee, Taegeon Oh, Dongjoon Lee, Changhwan Kim, Min Cheol Han, Chae‐Seon Hong, Jin Sung Kim

**Affiliations:** ^1^ Department of Radiation Oncology Yonsei Cancer Center Heavy Ion Therapy Research Institute Yonsei University College of Medicine Seoul South Korea; ^2^ Department of Radiation Oncology Yonsei Cancer Center Heavy Ion Therapy Research Institute Seoul South Korea; ^3^ Oncosoft Inc Seoul South Korea

**Keywords:** ANZAI laser sensor, CIRT, respiratory‐gated radiotherapy

## Abstract

**Background:**

Respiratory‐induced tumor motion remains a significant challenge in carbon‐ion radiotherapy (CIRT), potentially compromising dose accuracy and escalating dose in nearby organs. Although belt‐based sensors are commonly used for gating, issues related to setup reproducibility and patient comfort increased the demand for laser‐based, non‐contact alternatives.

**Purpose:**

This study evaluates the performance of an ANZAI laser‐based respiratory gating system for potential use in this modality based on total delay measurements and assessment of the accuracy and signal robustness of the sensor under a broad range of simulated and clinical conditions.

**Methods:**

The total system delay was quantified through segmentation of the signal pathway into four steps: motion‐to‐waveform, waveform‐to‐gate, gate‐to‐beam, and signal‐to‐irradiation. A programmable motion phantom was used to assess the tracking accuracy under various conditions, including changes in amplitude, period, and irregular breathing patterns, such as baseline shifts and phase variations. Signal robustness was evaluated with varying the sensor‐to‐surface distance (9–17 cm), sensor angle (0–45°), ambient lighting, and interference from adjacent lasers. A volunteer study correlated the external laser signal with internal diaphragm motion obtained via four‐dimensional computed tomography (4DCT) in supine and prone positions and assessed cross‐compatibility with a belt‐type sensor.

**Results:**

The total system delay was 59.3 ms for beam‐on and 40.4 ms for beam‐off, which is well below the 100‐ms threshold. In the phantom tests, the sensor achieved precise tracking, with cross‐correlation coefficients > 0.99 (regular and irregular respiratory patterns). The signal was robust across a 9–17 cm span and was stable in a dark environment. However, the measured amplitude increased at sensor angles > 20°, and considerable signal distortion occurred due to interference from an adjacent laser. A strong correlation between the sensor signal and internal organ motion was observed in volunteer tests in supine (*r* = 0.986) and prone (*r* = 0.961) positions. The interoperability with the belt sensor was excellent (*r* = 0.99).

**Conclusions:**

The ANZAI laser sensor system is accurate and reliable, and it meets clinical latency requirements in respiratory‐gated CIRT. It provides stable tracking under varied respiratory conditions. For optimal performance, maintaining a perpendicular sensor angle is recommended to ensure amplitude accuracy and mitigate crosstalk from adjacent lasers. These findings confirm the clinical readiness of the system and inform practical implementation.

## 1 INTRODUCTION

Carbon‐ion radiotherapy (CIRT) is an advanced radiation modality that provides a highly conformal dose distribution and enhanced relative biological effectiveness relative to conventional photon or proton therapies.^1^, ^2^, ^3^ Given these physical and radiobiological advantages, CIRT enables the delivery of ablative doses to malignant tumors while sparing adjacent healthy tissues.^4^, ^5^, ^6^, ^7^ However, treating tumors located in anatomically mobile organs, such as the lungs and liver, remains a major clinical challenge. Tumor displacement induced by respiration during treatment can result in deviations from the planned dose distribution; this phenomenon is termed motion‐induced dose blurring.^8^, ^9^, ^10^, ^11^ This effect is especially pronounced in pencil‐beam scanning CIRT, where a temporal mismatch between the scanning beam and the moving tumor generates an interplay effect, leading to localized hot or cold spots within the target volume.^12^ Without mitigation, this dosimetric uncertainty may compromise tumor control and increase the radiation dose to surrounding organs at risk.

Two principal motion mitigation strategies are used in clinical particle therapy to address these issues: respiratory gating and rescanning. Both techniques mitigate motion‐induced dose inhomogeneity.^13^, ^14^, ^15^, ^16^ More recently, a hybrid technique termed “phase‐controlled rescanning” has been introduced. This method combines gating with rapid rescanning during a predefined phase of the respiratory cycle to suppress interplay effects, thus improving dose uniformity and target coverage, even under irregular breathing conditions.^17^, ^18^

The successful implementation of these strategies requires a respiratory monitoring system that captures accurate and reproducible breathing signals in real time.^19^, ^20^, ^21^ A reliable external respiratory signal is essential to synchronize beam delivery with the correct breathing phase and ensure geometric alignment between the beam and tumor position.^22^, ^23^, ^24^

The widely used respiratory monitoring approach to CIRT involves a belt‐based pressure sensor that detects abdominal motion during respiration.^25^ Nonetheless, this approach presents several practical limitations, including complex setup and calibration, potential discomfort caused by tight fixation, and interference with immobilization devices, such as thermoplastic shells, especially in prone positions. Furthermore, variations in belt position and tension can cause inconsistent signal quality across treatment sessions, compromising reproducibility.

To overcome these limitations, noncontact laser distance sensors are now in use. For instance, the ANZAI AZ‐733VI system uses a laser to track the surface motion of the chest or abdomen, thereby providing a real‐time respiratory waveform. This approach eliminates physical contact, reduces setup time, and enhances patient comfort while delivering consistent and high‐quality signals without mechanical interference. Given these advantages, our institution has implemented a laser‐based respiratory gating system. However, prior to clinical deployment, its performance must be validated. Although the majority of studies have reported commissioning results primarily for pressure‐based sensors, limited data exist on the quantitative performance and quality assurance (QA) of laser‐based gating systems in carbon‐ion therapy.

This study aims to establish QA criteria for safe and accurate implementation of laser‐based respiratory gating in carbon‐ion therapy. Therefore, the physical performance of a laser‐based respiratory gating system in a CIRT environment was evaluated quantitatively. We assessed the signal latency, waveform accuracy and precision, and signal stability of the system under varying environmental conditions and quantified the correlation between external respiratory signals and internal organ motion. Additionally, we conducted a comparison against a belt‐based system to assess its compatibility and ensure safe clinical use.

## 2 METHODS

### 2.1 Respiratory gating system (ANZAI laser sensor)

**FIGURE 1:**
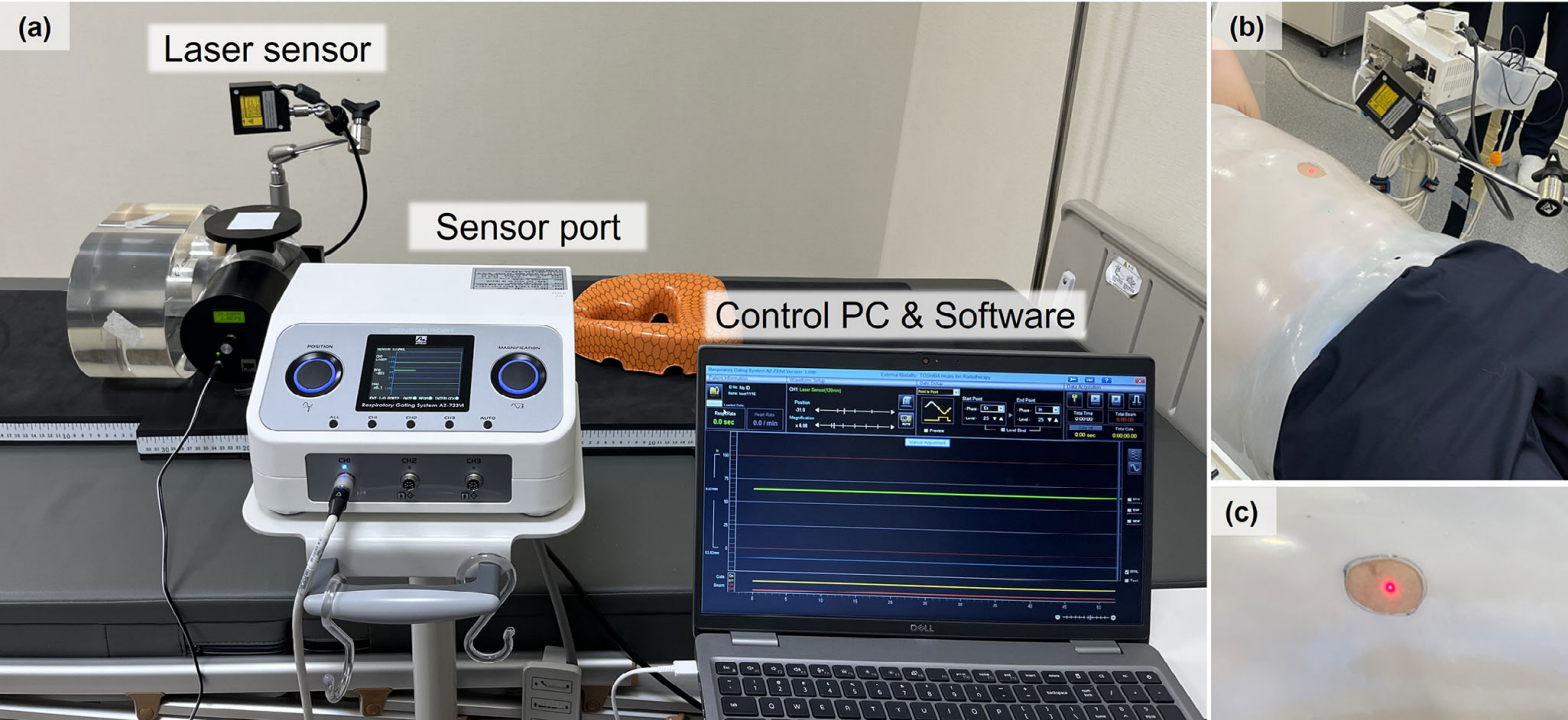
ANZAI laser‐based respiratory gating system and clinical setup with a thermoplastic immobilization shell. (a) System configuration depicting the laser sensor, sensor port, control personal computer (PC), and software interface. (b) Clinical setup with the laser sensor positioned above the shell for respiratory motion tracking. (c) Opening the shell allows unobstructed laser projection onto the patient's skin for accurate signal acquisition.

At our institution, an ANZAI laser‐based respiratory gating system (AZ‐733VI; Anzai Medical Co., Tokyo, Japan) was deployed for clinical use in CIRT to ensure accurate respiratory monitoring and gated beam delivery. The system comprised a laser sensor, sensor port, control personal computer (PC), and dedicated software (Figure 1a). This system directs a laser onto the patient's surface and analyzes the reflected signal, thus enabling real‐time respiratory motion tracking.

The gating system used the 120‐mm‐range laser sensor model, which features a measurement range of 120 ± 60 mm and a laser spot size of 1.0 × 1.5 mm. The sensor was operated with a 655 nm semiconductor laser with a maximum output of 1 mW. As the patient breathes, the sensor detects the displacement of the reflected laser signal caused by surface motion. This analog signal passed through a preamplifier to a control PC, where the software converted it into a real‐time waveform. This software enabled user‐defined gating windows to be set based on normalized amplitude thresholds (ranging from 0 to 100). These gating parameters configured the PC application to issue a gating command. The command was sent via USB to the relay box (RB), forwarded to the sensor port (SP), and output at the selected port as a physical gating signal through the EXT.I/O cable (or optional RS‐232C). The treatment unit used this signal to synchronize the beam on/off with the respiratory phase within preset limits.

### 2.2 Laser‐based respiratory monitoring with thermoplastic shell

Thermoplastic shells are routinely used in CIRT to immobilize patients during treatment. The shell is wrapped around the abdomen to provide stability. In clinical practice, these shells primarily improve immobilization and setup reproducibility rather than eliminating respiratory motion. Even when a shell covers the thoracoabdominal region, residual tumor motion of several millimeters typically observed under free breathing. For example, Takahashi et al. reported lung tumor motion in CIRT candidates with a mean amplitude of approximately 7 mm and a maximum of 22 mm despite the use of a thermoplastic shell.^18^

However, this setup obstructs respiratory motion monitoring of the thoracic or abdominal surface, making reliable surrogate signal acquisition challenging. To overcome this limitation, an aperture was created in the shell (Figure 1b, c). This opening allows unobstructed laser projection onto the skin surface for accurate detection of respiratory motion.

### 2.3 Response (Delay) time measurement

The latency of the ANZAI laser‐based respiratory gating system was evaluated by measuring each stage of signal transmission from respiratory motion detection to beam delivery. The process was partitioned into four sequential steps.

**FIGURE 2:**
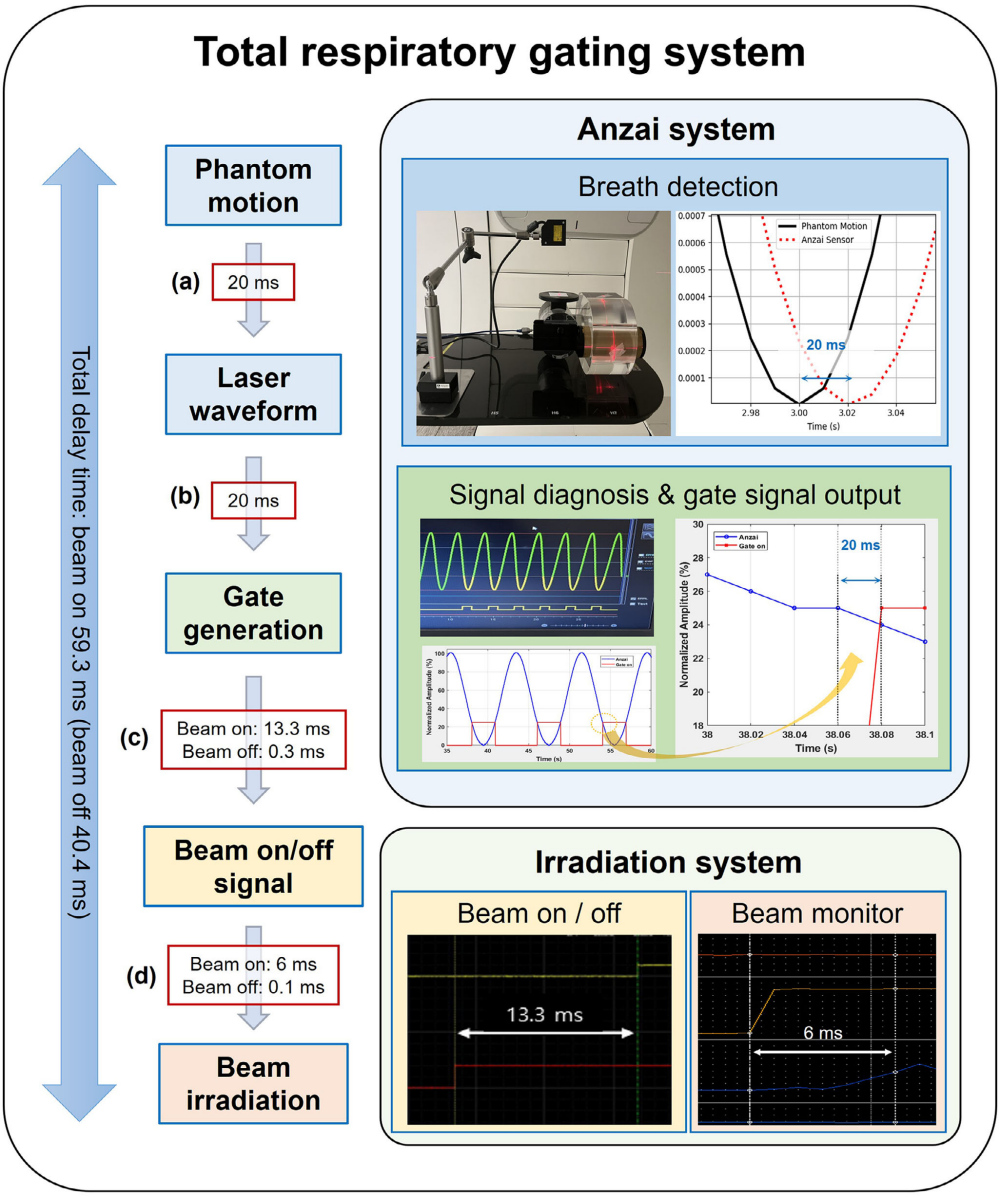
Schematic representation of latency measurement in the ANZAI laser‐based respiratory gating system. (a) Delay from phantom motion to waveform detection, (b) waveform to gate signal generation, (c) gate signal to beam‐on/off signal, and (d) beam‐on/off signal to actual beam irradiation. The total system delay was 59.3 ms for beam‐on and 40.4 ms for beam‐off.

First, the delay between the actual motion and waveform generation (Figure 2a) was measured using a motion phantom (Quasar Motion Phantom, Modus Medical Devices Inc., London, Canada) programmed with a sinusoidal respiratory pattern. The phantom input signal served as a reference and was compared against the waveform acquired by the laser sensor. Following the alignment of the initial peaks, the average time difference between the subsequent peaks was calculated.

Second, the latency between the waveform signal and the generation of the gate signal (Figure 2b) was measured. The waveform was continuously monitored to determine when it fell within the predefined gate window based on the amplitude thresholds (e.g., lower level 0 and upper level 20). Using ANZAI software, the gate signal and the waveform were compared following alignment to a common time axis. The time difference between the waveform and gate signal was measured to quantify the latency at this stage. These phantom waveform and gating settings were configured to be consistent with the amplitude‐gating strategy intended for clinical CIRT under full‐shell immobilization at our institution, where the external surrogate is represented on a normalized amplitude scale from 0 to 100 and clinical gating windows of 0%–10% or 0%–20% are typically used.

Third, the latency between the gate signal and the beam‐on/off signal from the irradiation system (Figure 2c) was measured. An oscilloscope was used to calculate the precise time difference between the two signals. This step was essential for verifying the synchronization between the gating and beam control systems.

Finally, the latency between the beam‐on/off signal and actual irradiation (Figure 2d) was measured using a data logger connected to the analog output of the dose monitor. This configuration enabled accurate tracking of the time difference between the signal trigger and actual beam delivery.

Each delay was measured independently and summed to calculate the aggregate latency. This systematic approach enabled a detailed analysis of the temporal characteristics of the ANZAI laser‐based respiratory gating system, thereby ensuring accurate synchronization between the respiratory motion and beam delivery.

### 2.4 Motion phantom test

#### 2.4.1 Verification of respiratory signal measurement accuracy

**FIGURE 3:**
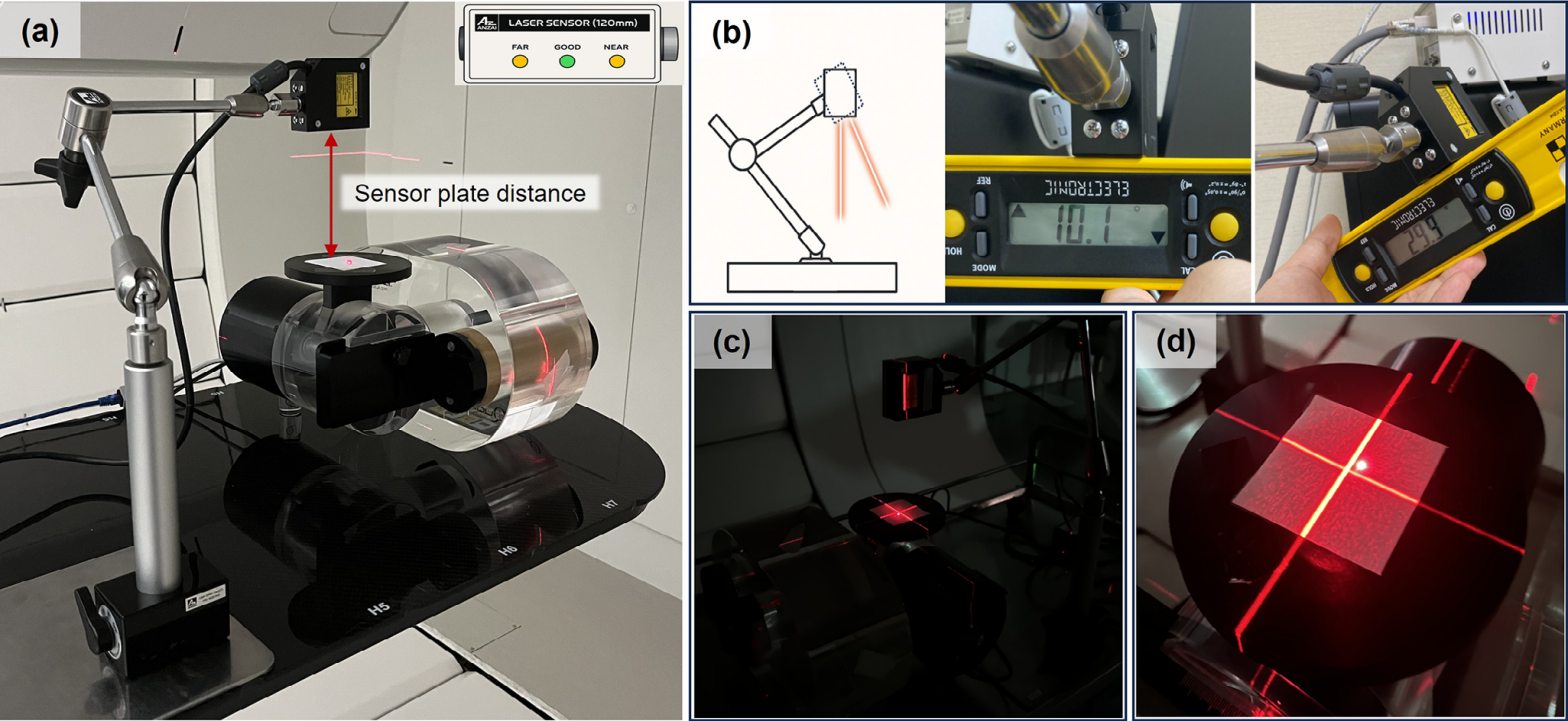
Motion phantom configuration for assessing the accuracy and robustness of the ANZAI laser sensor system. (a) Setup for validating respiratory signal acquisition under varying breathing conditions, including changes in period, amplitude, baseline shift, and irregular phase patterns. (b) Quantitative assessment of measurement accuracy at non‐perpendicular sensor angles. (c) Assessment of laser signal stability under low‐light (dark) conditions. (d) Assessment of signal interference from adjacent laser sources.

The tracking accuracy of the ANZAI laser sensor for the respiratory motion was assessed using a programmable motion phantom (Figure 3a). The phantom was programmed to generate respiratory motion signals under multiple conditions, which served as reference data for evaluation. These conditions included regular and irregular breathing patterns and enabled a comprehensive assessment of the sensor performance. The respiratory motion signals were configured with variable parameters to simulate different breathing conditions.

The key variables included amplitude, period, and irregularity, which were systematically modified to assess the tracking accuracy. Amplitude variation was assessed at 7.5 and 2.5 mm with the cycle fixed at 4 s. In our motion phantom, the maximum controllable travel of the target insert was 30 mm. Because the external motion plate was mechanically linked to the insert with a 1:4 travel ratio, the maximum validated peak‐to‐peak amplitude of the plate was 7.5 mm, which limited the evaluation of larger excursions at the plate level. To relate these amplitudes to clinical conditions, we referred to published statistics on external respiratory motion. Quirk et al.^26^ reported mean peak‐to‐peak abdominal excursions of 10.0 ± 5.0 mm in thoracic and abdominal cancer patients, 8.0 ± 3.0 mm in the abdomen of healthy volunteers, and 2.0 ± 2.0 mm at the thoracic wall in volunteers. In addition, Takahashi et al.^18^ reported a mean respiratory tumor motion amplitude of approximately 7 mm in lung cancer patients who were candidates for CIRT. Based on these reports, we considered 7.5 mm as a representative amplitude for abdominal motion and 2.5 mm as a representative value for small motion in this study.

For period variation, durations of 5 and 3 s were tested with a fixed amplitude of 5 mm. In addition to regular conditions, two irregular breathing patterns were modeled. The first was a baseline shift, in which the baseline respiratory signal drifted over time. The second involved irregular periods with random fluctuations applied to the signal periodicity. These irregular conditions were intended to mimic clinically relevant breathing variations. During the tests, the ANZAI laser sensor was placed to directly measure the surface motion of the phantom. The tracking accuracy was evaluated by comparing the sensor signals with reference motion data from the phantom. The agreement between the measured and reference signals was assessed using a cross‐correlation analysis. The cross‐correlation coefficients between the reference and measured signals were computed for each condition. A high cross‐correlation coefficient indicates strong agreement between the signals, thereby confirming the high tracking accuracy of the ANZAI laser sensor under diverse conditions.

#### 2.4.2 Signal robustness according to the measurement environment

The stability of the laser sensor signal was evaluated under multiple clinically relevant conditions to assess its stability and reliability. The tests were designed to evaluate signal stability and accuracy across variations in sensor distance, sensor angle, ambient lighting, and interference from adjacent lasers (Figure 3b, c). All sensor‐to‐surface distances were measured from the front face of the sensor housing. To assess distance‐related robustness, we used a center‐out staircase protocol at the nominal distance (120 mm). Starting at 120 mm, the distance was adjusted in 1 cm increments toward and away from the surface. In our setup, attempted distances thus spanned from ≤ 8 to ≥ 18 cm. However, stable automatic adjustment (a function that automatically normalizes the waveform level to the 0–100 range when the patient's respiratory amplitude is constant) was achieved only between 9 and 17 cm. At ≤ 8 or ≥ 18 cm, cross‐correlation was not evaluated, as the waveforms were unstable. The sensor was securely mounted, and the distance was finely adjusted using a movable stage. The programmable motion phantom generated a sinusoidal reference (amplitude 5 mm; period 4 s) that was continuously recorded by the laser sensor, and the light‐emitting diode (LED) alignment indicators (Near, Good, and Far) were monitored to confirm proper alignment. The recorded signals were then compared with the phantom input using cross‐correlation to assess signal stability.

To assess the effect of sensor alignment, the laser sensor was positioned at various angles ranging from 0 to 45° relative to the surface. The sensor was aligned at each angle, and the phantom produced a consistent signal (amplitude = 5 mm, period = 4 s). The sensor output was recorded and compared with the phantom input signal to assess measurement fidelity. To assess the effect of ambient light, the sensor was tested in dark conditions. The room lights were turned off to enable accurate measurements under low‐light conditions. The sensor was positioned at a standard distance, and the phantom produced a reference signal. The sensor output was recorded under dark conditions to assess the ability of the sensor to maintain accurate signal detection free of interference from external light sources. Finally, the effect of adjacent laser interference on the signal integrity was evaluated. Although uncommon, respiratory monitoring lasers may be placed near room‐alignment lasers in clinical settings. To simulate this, the laser point of the sensor was positioned adjacent to an active room alignment laser, and the signal output was continuously recorded. This setup enabled the evaluation of signal stability and accuracy under interference from lasers.

### 2.5 Volunteer test

#### 2.5.1 Correlation between external respiratory signal and internal organ motion at different postures

**FIGURE 4:**
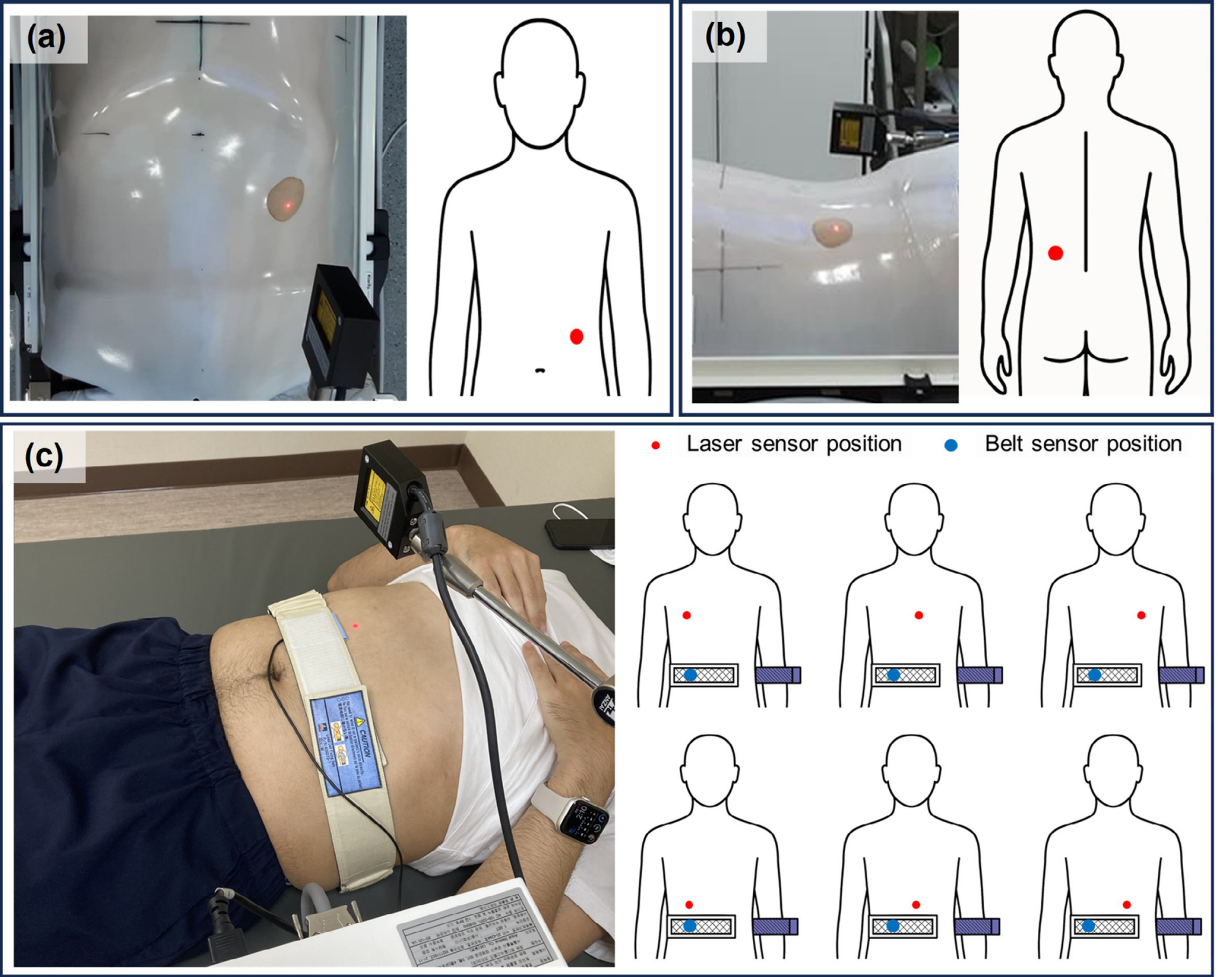
Volunteer test setup for assessing sensor positioning and cross‐compatibility in respiratory monitoring. (a) Laser sensor configuration in the supine position. (b) Laser sensor setup in the prone position, where the sensor detects respiratory motion on the posterior surface. (c) Test assessing the cross‐compatibility of laser and belt sensors by placing the sensor at various positions to determine the feasibility of substitution in clinical situations.

This test aimed to evaluate the ability of the ANZAI laser sensor to reliably detect external respiratory surrogate signals under conditions resembling clinical CIRT. In CIRT, an immobilization shell is routinely used to maintain stable patient positioning throughout treatment; however, this immobilization may affect natural breathing patterns by restricting surface motion. Therefore, we performed this test to evaluate the clinical validity of external respiratory signals measured under these immobilization conditions. We assessed the outcomes in the supine and prone positions. Although the prone position is less commonly used in clinical practice, it may be required in particular treatment scenarios. However, in the prone position, the patient's chest and abdomen are in close contact with the treatment couch, potentially reducing the external surface motion and raising uncertainty about the reliability of the respiratory measurements. Therefore, we specifically tested whether the ANZAI laser sensor could provide reliable respiratory signals in the prone position compared with the standard supine position. The experiment was conducted with an immobilization shell to replicate actual treatment conditions (Figure 4a, b).

The ANZAI laser sensor was positioned appropriately for each position to ensure accurate alignment of the laser beam with the measurement area. Abdominal surface motion was tracked in the supine position, whereas posterior torso surface motion was tracked in the prone position. External respiratory signals were continuously recorded as time‐series waveforms representing respiratory surrogate motion, thereby enabling the subsequent assessment of signal accuracy and correlation with internal organ motion. Four‐dimensional computed tomography (4DCT) was reconstructed using external respiratory surrogate signals. From the reconstructed 4DCT images, the diaphragmatic motion was extracted during multiple respiratory phases. The diaphragm displacement was then compared with the external signal measured by the ANZAI laser sensor to assess the signal correlation. Both signals were normalized and phase synchronized from 0% to 100% to enable accurate matching. Cross‐correlation analysis was used to quantify the agreement between the external signal and diaphragmatic motion for each position. This analysis assessed whether the ANZAI laser sensor accurately reflected the motion of the diaphragm in the supine and prone positions.

#### 2.5.2 Cross‐compatibility evaluation between the laser and belt sensors

This volunteer test was conducted to evaluate whether respiratory signals from the ANZAI laser sensor and a belt‐type respiratory sensor can be used interchangeably in clinical practice. A high correlation was expected when the two sensors monitored the same or a similar anatomical region. However, in actual treatment settings, the placement of the sensors can be constrained, and it is not always possible to position both sensors in an ideal location. Therefore, this test aimed to assess how well the cross‐compatibility between the two sensors is maintained under different placement configurations.

Cross‐compatibility was evaluated in three healthy volunteers. Each volunteer was instructed to perform abdominal breathing in the supine position, and the laser and belt sensors were positioned according to the configurations shown in Figure 4c. For each volunteer and each configuration, respiratory signals from both sensors were recorded simultaneously for approximately 3 min. Cross‐correlation analysis was then performed on the paired laser and belt signals to evaluate the level of agreement between the two systems.

## 3 RESULTS

### 3.1 Measurement of system delay time

The latency of the ANZAI laser‐based respiratory gating system was measured in four distinct stages, each representing a specific signal‐processing pathway in the gating system (Figure 2). In the first stage, the delay between the actual motion (modeled with a motion phantom) and the waveform signal detected by the laser sensor was measured. The waveform showed a delay of 20.0 ms from the initial motion input (Figure 2a). In the second stage, the delay between the waveform and the gate signal generation was measured. The gate signal was triggered when the waveform entered the predefined amplitude‐based gating window (lower level 0 and upper level 20), resulting in a delay of 20.0 ms (Figure 2b). In the third stage, the delay between the gate signal and the beam‐on/off signal from the irradiation system was measured. This delay was measured as 13.3 ms for beam‐on and 0.3 ms for beam‐off (Figure 2c). In the final stage, the delay between the beam‐on/off signal and actual beam irradiation was measured. This delay was measured as 6.0 ms for beam‐on and 0.1 ms for beam‐off (Figure 2d). The aggregate latency was calculated by summing all four stages, yielding 59.3 ms for beam‐on and 40.4 ms for beam‐off.

### 3.2 Validation of respiratory tracking accuracy with motion phantom

**FIGURE 5:**
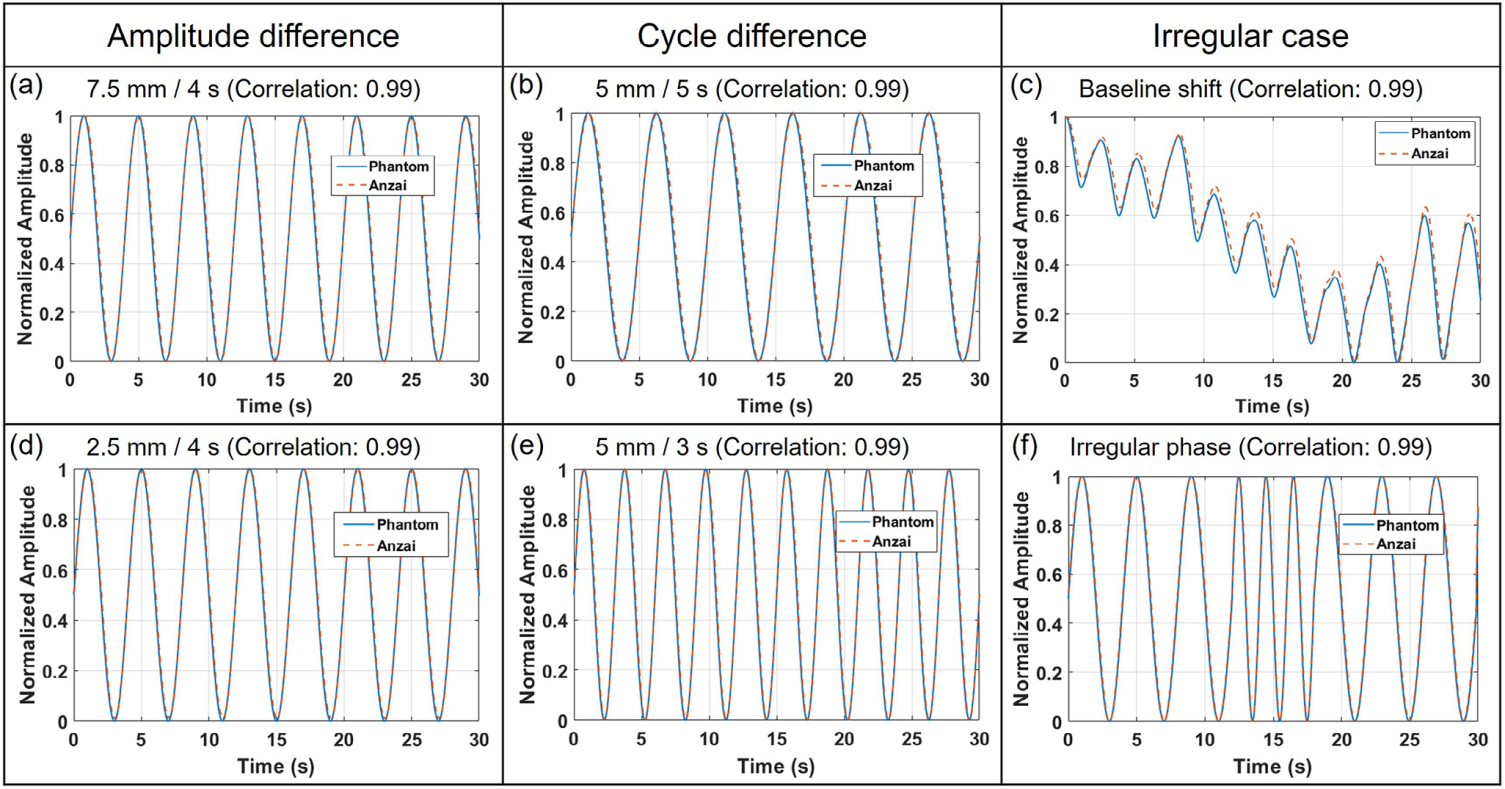
Reliability evaluation of the ANZAI laser sensor system with a programmable motion phantom. To evaluate the tracking accuracy of the sensor, respiratory motion signals were acquired under various conditions, including changes in amplitude, period, and irregular breathing patterns, such as baseline shifts and phase irregularity. The agreement between phantom motion and sensor measurements was quantitatively assessed using cross‐correlation coefficients for each condition.

The tracking accuracy of the ANZAI laser sensor for the respiratory motion was evaluated with a programmable motion phantom under various respiratory conditions (Figure 5).

To evaluate the effect of amplitude variation, the sensor was tested at two amplitudes (7.5 and 2.5 mm) with a fixed respiratory cycle of 4 s (Figure 5a, d). The cross‐correlation coefficients were 0.9907 (7.5 mm) and 0.9908 (2.5 mm), indicating high agreement between the sensor and reference signals. The effects of respiratory cycle variation were also assessed at 5 and 3 s, with the amplitude fixed at 5 mm (Figure 5b, e). The cross‐correlation coefficients were 0.9916 (5 s) and 0.9913 (3 s), indicating strong consistency across varied respiratory frequencies. In addition to regular patterns, the sensor performance was assessed under irregular conditions to simulate patient respiration. For the baseline shift condition (Figure 5c), in which the respiratory signal baseline drifted, the cross‐correlation coefficient was 0.9981. For the irregular phase condition (Figure 5f), in which the respiratory cycle varied randomly, the cross‐correlation coefficient was 0.9941. These findings confirm that the ANZAI laser sensor achieves high measurement fidelity under diverse respiratory conditions, including variations in amplitude, cycle duration, and irregular breathing patterns.

### 3.3 Evaluation of sensor robustness under geometric and environmental variations

Using a center‐out staircase with 1 cm steps around the nominal distance of 120 mm, we tested distance‐related robustness from ≤ 8 to ≥ 18 cm. Stable automatic adjustment was obtained only between 9 and 17 cm. The phantom generated sinusoidal motion with an amplitude of 5 mm and a period of 4 s. Sensor alignment was monitored using the built‐in LED indicator (Near, Good, and Far). At each distance, the sensor signal was compared with the phantom reference, and agreement was quantified using cross‐correlation (Figure 3a; Table 1). The LED showed Near at ≤ 11 cm, Good at 12–15 cm, and Far at ≥ 16 cm. Across the stable band of 9–17 cm, agreement remained consistently high without observable degradation. At ≤ 8 or ≥ 18 cm, a stable waveform could not be acquired; thus, cross‐correlation was not performed. All distances reported here are housing‐referenced, whereas the manufacturer's working range of 120 ± 60 mm is defined from the laser source point; absolute comparisons should therefore account for the difference in reference origin.

**TABLE 1:** Robustness of the laser sensor signal assessed at sensor‐to‐surface distances ranging from 9 to 17 cm.

Phantom input motion				
Amplitude [mm]	Cycle [s]	Sensor to motion plate distance [cm]	LED indicator of the sensor	Laser‐sensor measurement [Cross correlation with phantom motion]
5	4	≤ 8	Near [Yellow]	NA
		9		0.97
		10		0.97
		11		0.97
		12	Good [Green]	0.97
		13		0.98
		14		0.99
		15		0.97
		16	Far [Yellow]	0.98
		17		0.96
		≥ 18		NA

*Note*: Sensor‐to‐motion‐plate distances were measured from the front surface of the sensor housing. “Not Available (NA)” indicates that a stable waveform could not be acquired or maintained; thus, cross‐correlation was not evaluated. In these cases, automatic adjustment could not be enabled. Cross‐correlation coefficients between the phantom input motion and laser sensor signals were computed. LED status indicators (Near, Good, and Far) were recorded to reflect alignment accuracy.

Abbreviation: LED, light‐emitting diode.

The accuracy of the ANZAI laser sensor was assessed by varying the sensor‐to‐surface angle from 0 to 45°. The phantom was programmed to produce a consistent motion pattern with an amplitude of 5.0 mm and a period of 4.0 s. At each angle, the sensor‐measured amplitude and period were compared with the phantom input data (reference values). The agreement between the sensor and reference signals was quantitatively assessed via cross‐correlation analysis (Table 2). At the reference angle of 0°, the measured amplitude was 4.940 ± 0.015 mm, the period was 4.000 ± 0.005 s, and the cross‐correlation coefficient was 0.99078. For sensor angles of 5°, 7°, 10°, and 15°, the measured amplitudes remained close to 5 mm, exhibiting negligible departures from the reference condition. However, at angles of ≥ 20°, the measured amplitude progressively rose, with values of 5.157 ± 0.002 mm at 20°, 5.578 ± 0.004 mm at 30°, and 6.170 ± 0.003 mm at 45°. The period remained consistent at 4.000 ± 0.005 s across all angles. Under all test conditions, the cross‐correlation coefficient, calculated using the normalized amplitude data, remained above 0.99, implying negligible departures from the reference.

**TABLE 2:** Assessment of laser sensor tracking accuracy under varying sensor‐to‐surface angles (0 to 45°).

		Phantom motion		Laser‐based measurement		
Condition	Sensor angle (°)	Amplitude [mm]	Cycle [s]	Amplitude [mm]	Cycle [s]	Correlation
Reference	0	5.0	4.0	4.940 ± 0.015	4.000 ± 0.005	0.99
Angle dependency	5	5.0	4.0	4.905 ± 0.004	4.000 ± 0.001	0.99
	7			4.902 ± 0.004	4.001 ± 0.008	0.99
	10			4.990 ± 0.026	4.001 ± 0.009	0.99
	15			4.979 ± 0.007	4.000 ± 0.004	0.99
	20			5.157 ± 0.002	4.000 ± 0.005	0.99
	30			5.578 ± 0.004	4.000 ± 0.005	0.99
	45			6.170 ± 0.003	4.000 ± 0.005	0.99

*Note*: The measured amplitude and period were compared with the programmed phantom motion to assess angle‐dependent performance. Correlation coefficients quantified concordance.

**FIGURE 6:**
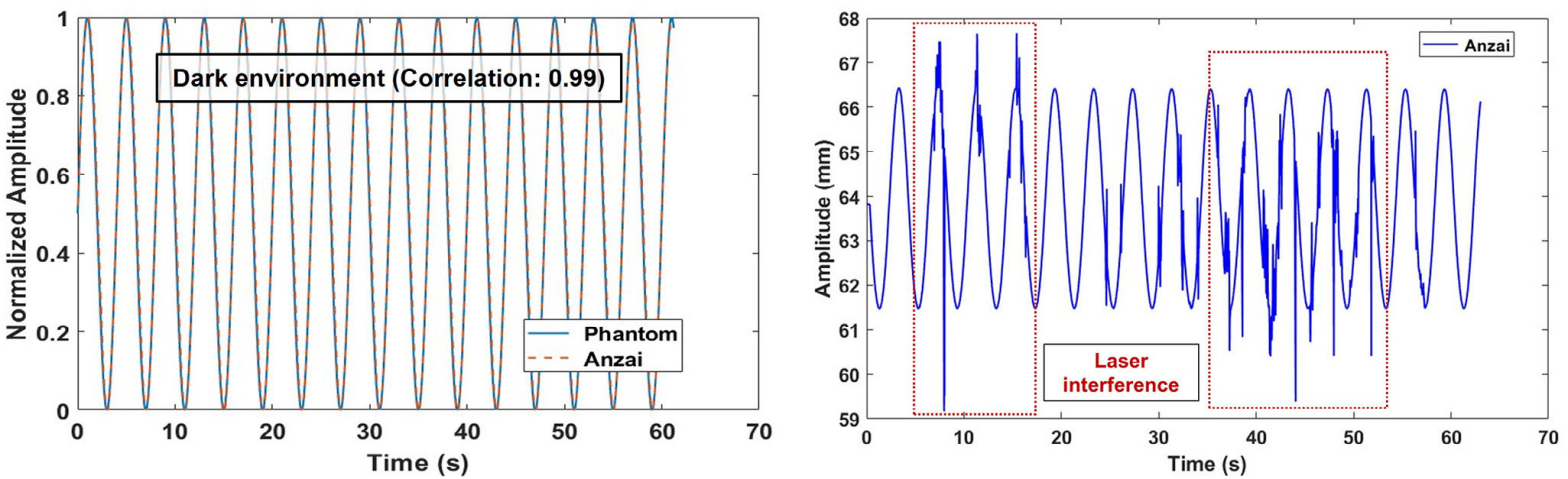
Signal robustness of the ANZAI laser sensor under external interference scenarios. Output signals measured in a completely dark environment (left) and under interference from a nearby room alignment laser (right).

The effects of ambient lighting and adjacent laser interference on ANZAI laser sensor signals were assessed under controlled conditions. The ambient‐light test was conducted in an entirely dark environment (Figure 3c), and the results are shown in Figure 6 (left).

Under these conditions, the acquired waveform remained stable and closely mirrored the reference signal with a cross‐correlation coefficient of 0.996. In contrast, signal instability occurred under adjacent laser‐interference conditions. When the room‐alignment laser was positioned near the laser point of the ANZAI sensor, the recorded signal exhibited irregular distortions and noise, as shown in Figure 6 (right).

### 3.4 Volunteer test

**FIGURE 7:**
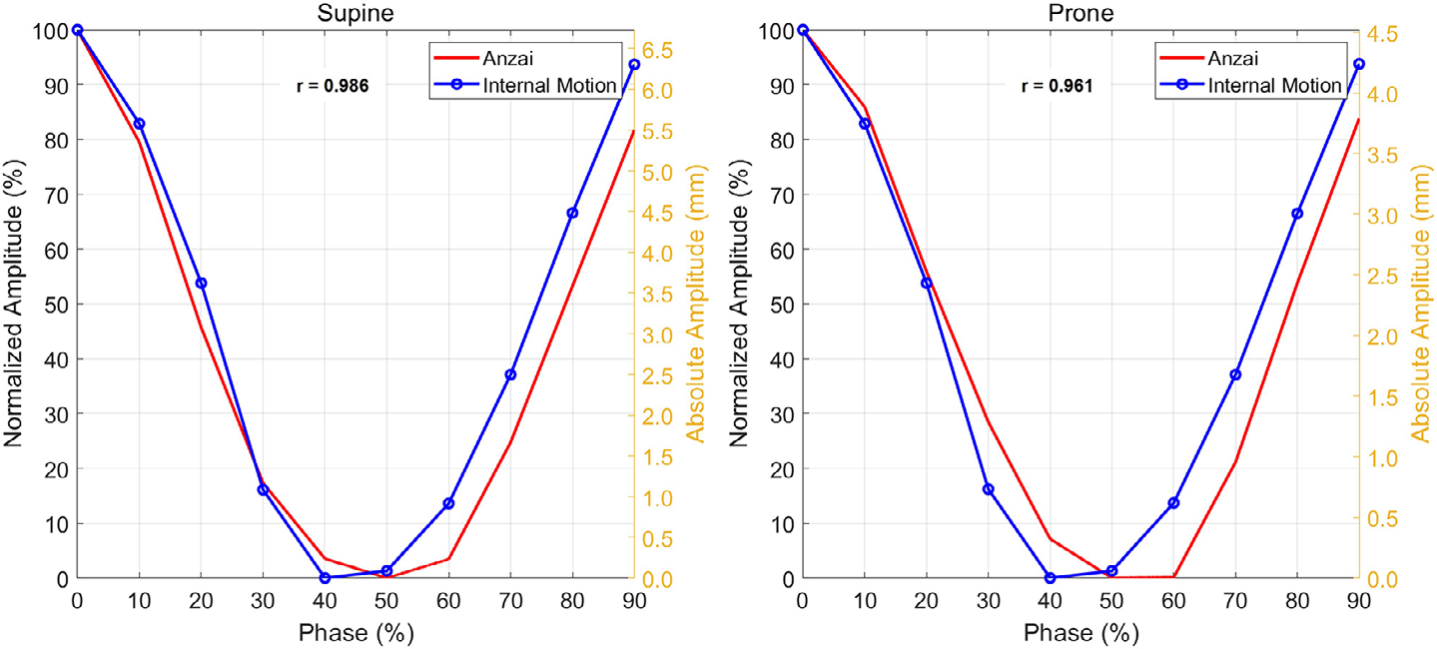
Comparison of external respiratory signals and internal organ motion by posture. For supine (left) and prone (right), the ANZAI laser sensor (red) and four‐dimensional computed tomography (4DCT)‐derived internal motion (blue) are displayed as normalized amplitude on the left y‐axis (0%–100%) and absolute amplitude on the right y‐axis (mm). Cross‐correlation coefficients (*r*) summarize concordance between the two signals.

The accuracy of the ANZAI laser sensor in tracking internal organ motion was evaluated in a single volunteer using an immobilization shell. External respiratory signals recorded by the ANZAI sensor were contrasted with internal motion reconstructed from 4DCT. For visualization, both signals are shown with normalized amplitude on the left y‐axis (0%–100%) and absolute amplitude on the right y‐axis (mm), and they are phase‐aligned from 0% to 100%. Agreement was quantified by cross‐correlation. As shown in Figure 7, the normalized amplitude curves from the ANZAI sensor and the 4DCT‐derived internal motion showed substantial similarity in both positions. In the supine orientation (Figure 7, left), the cross‐correlation coefficient was 0.986. In contrast, in the prone position (Figure 7, right), it was 0.961, indicating strong concordance between the external signal and internal motion for this representative case. Relative to the supine position, the prone position showed a modest reduction in peak absolute surface amplitude (approximately 2.1 mm, about a 32% decrease), although waveform morphology remained similar.

**FIGURE 8:**
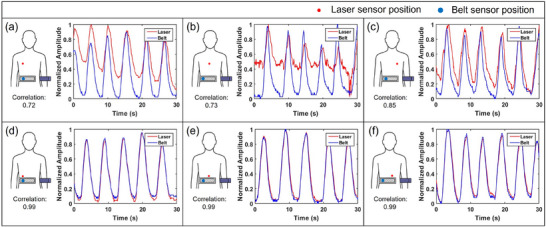
Comparison of respiratory signals acquired simultaneously by the laser sensor (red dots) and belt sensor (blue dots) placed at three positions on the chest and three positions on the abdomen. In each graph, the red and blue lines depict the laser and belt sensor signals, respectively. Correlation coefficients appear at the bottom of each panel to quantify the signal agreement for each configuration.

Figure 8 shows respiratory waveforms acquired simultaneously by the ANZAI laser and belt sensors for one representative volunteer (Volunteer 1) under six positioning configurations. This example illustrates how sensor placement influenced the agreement between the two signals. Table 3 summarizes the corresponding cross‐correlation for all three healthy volunteers. When the belt sensor was fixed at the right abdomen and the laser sensor was placed at the three chest positions, that is, relatively far from the belt, the correlation coefficients ranged from 0.73 to 0.86, with an overall mean of approximately 0.79. In contrast, when both sensors monitored the abdomen at three nearby positions, all volunteers showed consistently strong agreement, with correlation coefficients between 0.95 and 1.00 and an overall mean of approximately 0.98.

**TABLE 3:** Cross‐correlation between ANZAI laser and belt respiratory signals in three healthy volunteers.

Correlation efficiency between belt and laser sensor						
Belt sensor position	Right abdomen					
Laser sensor position	Chest region			Abdominal region		
	Right (a)	Center (b)	Left (c)	Right (d)	Center (e)	Left (f)
Volunteer 1	0.73	0.73	0.85	0.99	0.99	0.99
Volunteer 2	0.80	0.81	0.79	0.98	0.98	0.97
Volunteer 3	0.75	0.82	0.86	0.95	1.00	0.99

*Note*: The belt sensor was fixed at the right abdomen, and the laser sensor was placed at three positions on the chest and three on the abdomen (right, center, and left), corresponding to the configurations shown in Figure 8a–f.

## 4 DISCUSSION

### 4.1 Measurement of system delay time

Mizuno et al.^25^ measured the delay between the motion input and waveform generation using a laser range meter, thereby providing a benchmark. This approach assumes zero inherent delay in the laser sensor, yielding easier quantification. However, laser sensors inherently exhibit small but detectable latencies due to light propagation and internal signal processing. Neglecting this delay can underestimate overall latency. In contrast, this study quantified per‐component latency in the ANZAI laser‐based respiratory gating system, including the laser sensor. This approach permits precise evaluation of latency at every transmission step, ensuring high reliability when calculating the total system delay. The total system delay measured in this study (59.3 ms for beam‐on and 40.4 ms for beam‐off) fell comfortably below a 100 ms threshold specified in the Physical and Technological OA guidelines for particle beam therapy. The measured delay times also met the tolerance value of 100 ms set in the Task Group (TG)‐142 report of the *American Association of Physicists in Medicine*. These results confirm that the ANZAI laser‐based gating system can reliably synchronize respiratory motion with beam delivery, thus enabling precise, reliable delivery.

### 4.2 Validation of respiratory tracking accuracy with motion phantom

The results shown in Figure 5 show that the ANZAI laser sensor maintained a consistently high accuracy across a range of respiratory conditions. In all the test scenarios, the cross‐correlation coefficient exceeded 0.99, thus evidencing robust repeatability and dependability.

In particular, the irregular phase condition included challenging scenarios in which the respiratory cycle was shortened to approximately 1 s and the motion amplitude was reduced to approximately 1 mm. Even under these challenging conditions, the ANZAI laser sensor maintained stable tracking performance. This outcome indicates that the sensor offers accurate, sensitive, and fast detection of subtle and rapid respiratory variations.

### 4.3 Evaluation of sensor robustness subject to geometric and environmental variations

According to Fayad et al.,^27^ the breathing‐driven displacement of the abdominal and thoracic surfaces is 18.1 and 12.3 mm on average, respectively. Thus, the typical chest and abdominal movements caused by respiration can be adequately captured within the operational range of the ANZAI laser sensor used in this study (±60 mm, total of 120 mm). To optimize sensor performance, we recommend positioning the system so the breathing baseline coincides with the center of the operational range of the sensor (120 mm). This configuration ensures that the peaks of the respiratory signal remain within the effective measurement range (60–180 mm). Based on the measurement results, the LED status indicator on the sensor can be used as a reliable reference for consistent placement.

The results of the angular robustness evaluation showed that the cross‐correlation coefficient remained at values > 0.99 under all tested conditions, thus evidencing robust tracking fidelity relative to the reference orientation. Cross‐correlation analysis was performed using normalized amplitude data. The respiratory cycle was consistently measured at 4 s across all angles, signaling dependable timing. However, a trend emerged for amplitude estimates. Up to approximately 15°, the measured amplitude tracked the reference amplitude. However, at angles exceeding 20°, the measured amplitude progressively rose. This occurs because at steeper sensor angles, the laser sensor measures the diagonal distance between the sensor and the surface rather than the true perpendicular motion of the patient's surface, thereby inflating path length. Therefore, when precise absolute respiratory amplitude tracking is required (e.g., in quantitative motion studies), the sensor should be positioned as close to the skin as possible. In most other clinical scenarios, maintaining a consistent sensor angle between CT simulation and treatment suffices to preserve reliability and repeatability. In routine clinical gating, the external surrogate of the laser sensor is used on a normalized 0–100 scale; hence, delivery is governed by relative amplitude windows rather than absolute millimeter values. Consequently, angle‐related amplitude scaling does not degrade timing fidelity, and high correlation with the reference signal is maintained even at oblique angles. These findings are corroborated in Table 2.

The ANZAI laser sensor demonstrated stable signal performance, even in dark settings. Although these conditions are uncommon in clinical practice, the results suggest that accurate respiratory signal acquisition can be achieved during CT simulations or treatments in very dim rooms. However, care is warranted regarding adjacent laser interference. When the room‐alignment laser was positioned near the laser point of the ANZAI sensor, the recorded signal exhibited distortions plus noise artifacts. This indicates that overlapping or closely aligned room lasers can disrupt the accurate detection of respiratory signals. Therefore, during the sensor setup, operators must verify that room‐alignment lasers do not intersect or overlap with the measurement point of the sensor.

### 4.4 Volunteer test

In CIRT, the use of a thermoplastic immobilization shell is crucial for maintaining the patient's position. However, this setup may restrict the natural surface motion of the thorax or abdomen, limiting precise surrogate capture. To evaluate whether these signals can reliably represent internal organ motion under these conditions, we analyzed the cross‐correlation between the external waveforms measured by the ANZAI laser sensor and the motion of the diaphragm reconstructed from the 4DCT images. To the best of our knowledge, no prior studies have explicitly quantified the correlation between the external signals measured by the ANZAI laser sensor and the internal respiratory motion, precluding direct benchmarking of these results. According to Wang et al.,^28^ who evaluated the correlation between external respiratory signals (acquired with the Varian real‐time position management system) and internal lung and liver tumor motion (quantified via 4DCT), correlation strength can be classified as weak (*r* < 0.3), moderate (*r* = 0.30–0.69), or strong (*r* = 0.70–1.0). Although this study conducted an initial evaluation based on a single volunteer, which limits generalizability, the observed correlations were high (0.986 supine and 0.961 prone), suggesting promising clinical applicability of the ANZAI laser sensor as a respiratory surrogate. Although this initial internal‐motion analysis was based on a single volunteer and therefore has limited generalizability, these findings should be interpreted as proof‐of‐concept rather than a fully generalizable result, and validation in a larger cohort is required before broader clinical application. The prone position is less commonly used in clinical practice but may be necessary for particular cases. In this posture, the patient's chest and abdomen are in close contact with the treatment couch, which can reduce external surface motion and diminish surrogate amplitude. Despite this potential limitation, the ANZAI laser sensor showed strong concordance between external signals and internal motion in the prone position. Although the measured amplitude was slightly lower than that in the supine position, the results indicate that the ANZAI sensor delivers dependable tracking under reduced surface mobility conditions.

Another key consideration is the potential effect of shell modification on patient immobilization and setup reproducibility. A minor aperture was created in the upper abdominal region to enable direct laser projection onto the skin surface. This area was carefully selected because it did not contribute considerably to the structural rigidity of the shell, thereby limiting impact on immobilization and permitting robust surrogate capture. A similar approach was reported by Mizuno et al.,^25^ at another location. Additionally, a recent scoping review by Lastrucci et al.^29^ showed that open‐face thermoplastic masks used in head and neck radiotherapy provided immobilization accuracy and setup reproducibility comparable to those of conventional closed masks. Nevertheless, when these modifications are required in clinical practice, they require cautious implementation, considering patient‐specific anatomy and treatment conditions, and should be accompanied by a simple verification of setup reproducibility. This verification includes confirming that the shell fits similarly as in the CT simulation, checking the hole location and size and the sensor position (standoff within the validated range, incidence angle, and fixation), and performing image‐guided setup verification to confirm that patient position is within institutional tolerances (e.g., an orthogonal kV pair or cone‐beam computed tomography).

In our cross‐compatibility evaluation, the ANZAI laser sensor and the belt‐type respiratory sensor showed a clear dependence of signal agreement on their relative positioning. When the belt sensor was fixed at the right abdomen and the laser sensor was placed at the three chest positions, which were relatively distant from the belt, the correlation coefficients ranged from 0.73 to 0.86 with an overall mean of approximately 0.79. In contrast, when both sensors monitored the abdomen at three nearby positions, all volunteers showed consistently high agreement, with correlation coefficients between 0.95 and 1.00 and an overall mean of approximately 0.98. The small variation across volunteers and configurations in these abdominal placements indicates that stable and reproducible cross‐compatibility can be achieved when the two systems are positioned over adjacent anatomical regions. Taken together, these findings indicate that the relative positioning of the two systems is a key determinant of signal compatibility, and that when the sensors are placed at adjacent anatomical locations, they can provide respiratory surrogate signals that are effectively equivalent. From a clinical perspective, this suggests that even when the laser sensor cannot be used, belt‐based monitoring can still be employed in a compatible manner, provided that the belt sensor can be positioned over the region to be monitored. However, these observations were obtained from three healthy volunteers under instructed abdominal breathing in the supine position, and further studies in patient populations and under more diverse breathing conditions will be needed to fully generalize the cross‐compatibility between the two systems.

## 5 CONCLUSIONS

This study evaluated the performance of an ANZAI laser sensor for respiratory‐gated CIRT. The delay times measured at each stage of the signal transmission met clinical thresholds, confirming that the system is suitable for synchronization with the actual beam delivery timing. This sensor provides stable and accurate respiratory signals under various breathing conditions, including changes in the amplitude, period, and irregular patterns. Additionally, factors that may affect signal measurement in real‐world settings, including the sensor position, patient posture, ambient lighting, sensor angle, and interference from nearby lasers, were evaluated to identify the appropriate measurement conditions. A comparison with a belt‐type sensor was conducted, confirming the signal correlation between the two sensors and the conditions under which they can be used interchangeably. These findings provide actionable guidance that supports the flexible use of ANZAI laser sensors in real‐world clinical settings.

## AUTHOR CONTRIBUTIONS

Tae‐Ho Kim, Soorim Han, Dohyeon Yoo, Sangmin Lee, Min Cheol Han, Chae‐Seon Hong, and Jin Sung Kim contributed to the study conception and design. Tae‐Ho Kim, Taegeon Oh, and Dongjoon Lee conducted data collection. Tae‐Ho Kim and Changhwan Kim drafted and revised the manuscript. Changhwan Kim verified the authenticity of all raw data. All authors reviewed and approved the final manuscript.

## CONFLICT OF INTEREST STATEMENT

The authors declare that they have no competing interests.

## ETHIC STATEMENT

This study was approved by the Institutional Review Board (IRB, approval number: 4‐2025‐0847) of Yonsei University Hospital. All data were fully anonymized before the investigators accessed them.
